# The prevalence of malnutrition during admission to the pediatric intensive care unit, a retrospective cross-sectional study at Tikur Anbessa Specialized Hospital, Addis Ababa, Ethiopia

**DOI:** 10.11604/pamj.2022.41.77.31284

**Published:** 2022-01-27

**Authors:** Semhal Getachew Teka, Rahel Argaw Kebede, Charles Sherman

**Affiliations:** 1 Addis Ababa University, College of Health Sciences, Department of Pediatrics and Child Health, Addis Ababa, Ethiopia,; 2 Alpert Medical School of Brown University, Rhode Island, USA

**Keywords:** Stunting, wasting, pediatric intensive care unit, mechanical ventilation

## Abstract

**Introduction:**

malnutrition is a common problem in Ethiopia. Studies show malnourished children in intensive care units succumb more often to infection and death but Ethiopia has no available data to assess the prevalence of malnutrition in children admitted to pediatric Intensive Care Unit and their clinical patterns, this study was conducted to assess these variables in a tertiary hospital in Ethiopia.

**Methods:**

this was a retrospective cross sectional study done on 243 children, ranging from 1 month to 15 years of age, from January 2016 to December 2018. Anthropometric interpretation was done using WHO Z score charts. The assessed outcome variables were death, length of stay in pediatric intensive care unit (PICU), days on mechanical ventilator and hospital acquired infection. Collected data was entered and analyzed using SPSS 20.0 version.

**Results:**

the overall prevalence of wasting was 37.8% (n=92). Stunting was seen in 45.7% (n=111). Compared to well-nourished children, malnourished children were more likely to require mechanical ventilation (78.3% versus 66.2% OR-2, p=0.045), experience longer time on mechanical ventilation (10.3±13.2 days versus 6.1±7.9 days, p=0.012), develop hospital acquired infection (HAI) more often (30.4% versus 19.2%, p=0.045), and have a prolonged length of stay (10.7±16.4 days versus 6.1±8.4 days, p=0.005).

**Conclusion:**

malnutrition in our PICU was identified to be a common cause of morbidity associated with greater need for mechanical ventilation, prolonged ventilator days, increased HAI, and longer hospital stays. Taking into consideration scarcity of resources, malnutrition imposes great burden on clinical care.

## Introduction

According to the world health organization malnutrition is defined as the cellular imbalance between the supply of nutrients and energy and the body´s demand for them to ensure growth, maintenance, and specific functions. It is a state of deficiency or excess of energy, protein, and other nutrients [1]. There is high incidence of malnourished children in developing countries where it is known to be one of the most important contributors to child mortality. The effect of Malnutrition on disease severity and outcome in hospitalized children especially to those admitted to the intensive care unit is largely unaddressed [2,3].

Malnutrition and disease have an adverse relationship. The disease state may cause secondary malnutrition. In addition, malnutrition may contribute to delayed recovery or unfavorable outcomes in critically ill patients like delayed mechanical ventilation, mortality, longer length of stay and infection. Thus making assessment of nutritional status extremely important in patients who are admitted to the pediatric intensive care unit [4,5]. The adverse outcomes of critically ill patients in the pediatric intensive care unit might be attributed to the deterioration of nutritional status during their stay in ICU. This is due to different reasons that limit the provision of ideal nutrition, such as volume restrictions, procedures and interventions, frequent food breaks and lack of standardization of evidence-based processes for better nourishment. The different types of conditions and disorders they present with also plays a role in increasing the nutritional requirements that expose the patient to metabolic stress further aggravating the malnutrition by placing them at a higher risk of developing organ dysfunctions and several morbidities [6,7].

Nutritional status of patients during admission to the PICU can be assessed using clinical signs of malnutrition, biochemical indicators, and anthropometry. Anthropometry has an important advantage over other nutritional indicators because it is non-invasive, inexpensive, and relatively easy to obtain [8]. In this study the term malnutrition corresponds to under-nutrition and does not include the other form of it, obesity. This study is aimed to determine the prevalence of nutritional status at admission, as assessed by BMI for age, height for age and weight for height on the WHO Z-score and identify the effect of nutritional status on the patients´ outcome.

## Methods

The study was conducted at the pediatric intensive care unit of Tikur Anbessa Specialized Hospital, the largest teaching hospital under the administration of Addis Ababa University, College of Health Sciences.

**Study design and period:** the study employed a cross-sectional descriptive study design and was conducted among pediatric patients who were admitted to the PICU in Tikur Anbessa Specialized Hospital (TASH) from January 2016 to December 2018 GC. Data was collected by chart review using data abstraction form.

**Participants:** all patients admitted to the PICU, TASH for more than 24 hours were the source population. The study population was all patients admitted to the PICU from January 2016 to December 2018 who were between the ages of 1 month to 15 years for whom anthropometric parameters were available and did not leave against medical advice.

**The sample size:** it was calculated by using single population proportion formula. Due to absence of studies done in Ethiopia, the prevalence was considered to be 50% to calculate the sample size.

**Data collection:** chart numbers of patients admitted to the pediatric intensive care unit of Tikur Anbessa Specialized Hospital from January 2016 to December 2018 were retrieved from the pediatric intensive care unit Health Management Information System logbook. The selection was made according to inclusion and exclusion criteria and data from the patient charts was collected between May and June of 2019 by the investigator. Information was gathered by using a data abstraction form; socio-demographic, relevant anthropometric and other clinical data were retrieved

**Statistical analysis:** the principal investigator checked the data for its completeness and missing information during data collection and was entered into SPSS 20 manually. The result was summarized using percent, mean, and median. Associations were done by using the chi-square test for categorical variables and associations of continuous variables were done by using t-test. Associations are considered statistically significant when the p-value is less than 0.05 with confidence of interval of 95%.

**Ethical consideration:** ethical clearance was obtained from pediatrics and child health department´s research and publication committee of the school of medicine, college of health sciences at Addis Ababa University. A permission letter was provided to Tikur Anbessa Specialized Hospital chart documentation office to proceed with data collection. Anonymity was assured on the data retrieval form by omitting names and telephone numbers.

## Results

The results of the study are based on 243 patient charts. Patients mean age was 3.9 (SD 3.9) years, ranging from 1 month to 14 years. Majority (60.9%) of the patients were male. On the assessment of vaccination status, 15.4% of the patients were not vaccinated, 7.9% were partially vaccinated, and the rest were either fully vaccinated or vaccinated for their age. 77.8% of the patients were exclusively breastfed, 17.6% had mixed feeding (breast milk and formula milk), and 4.6% were not breastfed at all. Complementary feeding was started below the age of 6 months in 35.8% and at or above 6 months in 64.2% of the patients.

The frequency of primary disease category in decreasing order was: infectious diseases in 28.8%, disease of the nervous system in 22.6%, hematologic diseases in 14.8%, cardiac diseases in 13.5%, diseases of the respiratory system in 10.6%, and renal diseases in 5.7% respectively. As shown in Table 1, the prevalence of malnutrition on admission to the pediatrics ICU was found to be very common.

**Table 1:** nutritional status of the children at admission to pediatric ICU

		N (%)
WFH and BMI for age	Obese	5 (2%)
	Overweight	10(4.1%)
	Risk of overweight	12 (4.9%)
	**Wasted**	**47 (19.3%)**
	**Severely wasted**	**45 (18.5%)**
	Normal	124 (51%)
WFA	**Underweight**	**39 (18.8%)**
	**Severely underweight**	**57 (27.4%)**
	Normal	112 (53.8%)
HFA	**Stunted**	**54 (22.2%)**
	**Severely stunted**	**57 (23.5%)**
	Normal	132 (54.3%)

IUC: intensive care unit; BMI: body mass index; WFA: weight for age; HFA: height for age; WFH: weight for height;

The highest prevalence of malnutrition was seen in those patients below and equal to the age of one year with a prevalence of 39% of those patients with acute malnutrition on admission to the PICU. The overall prevalence of stunting was also highest in those patients below or equal to 01 years accounting for 41.4% of the patients who have to stunting on admission to the PICU. As depicted in Table 2, the majority of the patients admitted to the PICU required mechanical ventilation and had high mortality rates. The presence of malnutrition at admission to the PICU was associated with poor treatment outcomes as shown in Table 3.

**Table 2:** outcome of children admitted to PICU

Variables		N (%)
Mechanical ventilation	Yes	172 (70.8%)
	No	71 (29.2%)
HAI	Yes	57 (23.5%)
	No	186 (76.5%)
Outcome at discharge from PICU	Alive	118 (48.6%)
	Died	125 (51.4%)

HAI: hospital acquired infection; PICU: pediatric intensive care unit

**Table 3:** the effect of malnutrition on the outcome of children admitted to PICU

		WFH				
		Normal		Wasted		
		N	%	N	%	P value
MV	Yes	100	66.2%	72	78.3%	0.045
	No	51	33.8%	20	21.7%	
HAI	Yes	29	19.2%	28	30.4%	0.045
	No	122	80.8%	64	69.6%	
Outcome	Died	73	48.3%	52	56.5%	0.226
	Alive	78	51.7%	40	43.5%	
		Mean	SD	Mean	SD	
Length of ICU stay (days)		6.1	8.4	10.7	16.4	0.005
Length of MV (days)		6.1	7.9	10.3	13.2	0.012

WFH: weight for height; MV: mechanical ventilation; HAI: hospital acquired infection ICU: intensive care unit

## Discussion

The overall prevalence of malnutrition during admission to the PICU based on the WHO growth curves in our study was found to be significant with 37.8% of the patients wasted and 45.7% stunted Table 1. This finding is comparable to the finding of a study done in Brazil that showed 45.5% of the admissions to the PICU was malnourished [9]. While it was slightly less than the studies done in Iran and India that found malnutrition in more than half, 55.1% and 57.2% respectively, of the patients during admission. When compared to a study done in Netherlands, a developed country that showed a 24% prevalence of the malnutrition during admission to the PICU our study has a higher prevalence [2,10].

Patients below and equal to the age of 01 year showed the highest prevalence of malnutrition accounting for 39% and 42.2% of those with severe malnutrition. These results are similar to the findings of an Indian study that found the average age for the severely malnourished group to be 9 months. The high prevalence of severe malnutrition at this age group according to the same research may be due to the practice of exclusive breastfeeding without proper weaning and supplementary nutrition in developing countries [2].

The overall mortality rate in this study is 51.4% (Table 2) which is comparable to an Egyptian study that found the overall death rate to be 50.49% [11]. In our study the presence of malnutrition was not associated (p value 0.226) with the rate of mortality which is also the case in an Indian research where they did not find any statistically significant relationship between mortality and malnutrition [2]. Malnutrition was not found to be a predictor of greater risk of mortality on the univariate analysis (P value 0.382) on another research done in Brazil [9].

According to our results, the need for mechanical ventilation was 78.3% in children with malnutrition compared to 66.2% without malnutrition which was statistically significant with a P-value of 0.045 and the length of stay on mechanical ventilation was increased in those with malnutrition with the mean stay being 10.3 in patients with malnutrition and 6.1 in patients with normal anthropometric measurements and showed significant association with a p-value of 0.012 Table 3. These findings correlate with an Indian data that analyzed ventilator days among the groups based on nutritional status and found that underweight children required prolonged ventilator support compared to normal children and overweight/ obese children. (OR-2, p-0.03) [8]. Similar findings are also seen in another study done in a developing country where Malnutrition was associated with greater length of ventilation on the multiple logistic regression model (OR 1.76, 95%; CI 1.08-2.88; P = 0.024). This association of Malnutrition and increase in length of ventilator support may be explained by the several adverse effects that malnutrition has on respiratory function [9]. In our study malnutrition prolonged the duration of stay in the PICU with a mean stay in children with malnutrition of 16.4 days compared to 6.1 days in children with normal nutritional status. This difference was statistically strong with a p-value of 0.005 Table 3. This result is similar to research from a developing country that showed malnourished children had higher odds for prolonged PICU stay compared to normal children (OR-2, p-0.03) [8]. This result was also seen in a Brazilian study where malnutrition was a risk factor for length of ICU stay on univariate analysis ( p-value-0.044) [9].

In this study, the incidence of HAI was found to be higher in children with malnutrition at admission to the PICU. More than thirty percent (30.4%) of the children with malnutrition developed HAI compared to 19.6% of the patients with normal anthropometric measurements (p-value 0.013) Table 3. The researcher was not able to find other papers with a similar result but this finding can be explained by the fact that this research found an increase in PICU stay in patients with malnutrition in addition to the effect of malnutrition; on the immune functions that make them susceptible to infections [2].

**Limitation of the study:** since the study was a chart review we were unable to extract a complete nutritional history and family income due to incomplete medical documentation.

## Conclusion

The prevalence of malnutrition at admission to the pediatrics ICU at Tikur Anbessa Specialized Hospital was found to be very common. The overall prevalence of wasting was 37.8%, underweight 46.2%, and Stunting in 45.7% of the children at admission to the pediatric ICU The presence of malnutrition at admission was associated with poor outcomes. It was associated with prolonged ICU stay and increased requirements for mechanical ventilation. Malnutrition was also associated with the difficulty of weaning from the mechanical ventilator as shown by increased duration of MV in this group of children.

### What is known about this topic

- *Malnutrition is a common problem in the developing world;*
- *Malnutrition and infection have complex interdependent relationship where the occurrence of one worsens the other;*
- *Critically ill Children with malnutrition have poorer outcomes than those with normal nutritional status*.

### What this study adds

- *Our research has found that there is a high burden of malnutrition at admission to the PICU at Tikur Anbessa Specialized Hospital;*
- *We found a significant relationship between malnutrition and increased risk of hospital acquired infections*.
